# Impact of Xpert MTB/RIF and decentralized care on linkage to care and drug-resistant tuberculosis treatment outcomes in Johannesburg, South Africa

**DOI:** 10.1186/s12913-018-3762-x

**Published:** 2018-12-17

**Authors:** Denise Evans, Tembeka Sineke, Kathryn Schnippel, Rebecca Berhanu, Caroline Govathson, Andrew Black, Lawrence Long, Sydney Rosen

**Affiliations:** 1 Health Economics and Epidemiology Research Office, Department of Internal Medicine, School of Clinical Medicine, Faculty of Health Sciences, University of the Witwatersrand, Johannesburg, South Africa; 2 Health Economics Unit, School of Public Health and Family Medicine, Faculty of Health Sciences, University of Cape Town, Cape Town, South Africa; 3 Division of Infectious Diseases, University of North Carolina, Chapel Hill, NC USA; 4 Department of Internal Medicine, School of Clinical Medicine, Faculty of Health Sciences, University of the Witwatersrand, Johannesburg, South Africa; 5 Department of Global Health, Boston University School of Public Health, Boston, MA USA

**Keywords:** Rifampicin resistance, South Africa, Time to treatment initiation, Decentralization, Xpert MTB/RIF, HIV positive, Mortality

## Abstract

**Background:**

In 2011, South Africa improved its ability to test for rifampicin-resistant TB (RR-TB) by introducing GeneXpert MTB/RIF. At the same time, the South African National TB program adopted a policy decentralized, outpatient treatment for drug resistant (DR-) TB. We aim to analyze the impact of these changes on linkage to care and DR-TB treatment outcomes.

**Methods:**

We retrospectively matched adult patients diagnosed with laboratory-confirmed RR-TB in Johannesburg from 07/2011–06/2012 (early cohort) and 07/2013–06/2014 (late cohort) with records of patients initiating DR-TB treatment at one of the city’s four public sector treatment sites. We determine the proportion of persons diagnosed with RR-TB who initiated DR-TB treatment and report time to treatment initiation (TTI) before and after the implementation of Xpert MTB/RIF roll-out in Johannesburg, South Africa. We conducted a sub-analysis among those who initiated DR-TB treatment at the decentralized outpatient DR-TB centers to determine if delays in treatment initiation have a subsequent impact on treatment outcomes.

**Results:**

Five hundred ninety four patients were enrolled in the early cohort versus 713 in the late cohort. 53.8 and 36.8% of patients were diagnosed with multi-drug resistant TB in the early and late cohorts, respectively. The proportion of RR-TB confirmed cases diagnosed by Xpert MTB/RIF increased from 43.4 to 60.5% between the early and late cohorts, respectively. The proportion who initiated treatment increased from 43.1% (*n* = 256) to 60.3% (*n* = 430) in the late cohort. Pre-treatment mortality during the early and the late cohort reduced significantly from 17.5 to 5.8% while lost to follow-up remained high.

Although TTI reduced by a median of 19 days, from 33 days (IQR 12–52) in the early cohort to 14 days (IQR 7–31) in the late cohort, this did not translate to improved treatment outcomes and we found no difference in terms of treatment success or on-treatment mortality for those that initiated without delay vs. those that deferred initiation.

**Conclusion:**

Pre-treatment mortality reduced significantly during late Xpert MTB/RIF coverage but there was no significant difference after treatment was initiated. Despite improvements there is still a significant diagnosis and treatment gap for patients diagnosed with RR-TB and improving treatment outcomes remains critical.

**Electronic supplementary material:**

The online version of this article (10.1186/s12913-018-3762-x) contains supplementary material, which is available to authorized users.

## Background

The World Health Organization (WHO) estimates that only 20% of the 580,000 people eligible for treatment of multi-drug resistant and rifampicin-resistant tuberculosis (MDR/RR-TB) globally in 2015 were enrolled in care [1]. In South Africa, a high-burden country for drug resistant (DR-) TB, 64% of the 19,613 laboratory diagnosed cases of RR-TB, including those with MDR-TB, were enrolled for treatment that year, indicating a large gap between diagnosis and treatment [1, 2].

Rapid diagnosis and timely treatment initiation are fundamental to control of the spread of DR-TB. To this end, the South African National TB program launched decentralized DR-TB services and the national use of Cepheid’s Xpert MTB/RIF in 2011. Decentralization of services allows patients with DR-TB to be treated as outpatients, increases the number of treatment facilities, reduces cost, and improves time to treatment initiation [3–5]. Xpert MTB/RIF is a rapid molecular test that provides same day diagnosis of TB and rifampicin susceptibility. By accelerating diagnosis, Xpert MTB/RIF reduces time to treatment initiation, thereby increasing the proportion of diagnosed patients who receive treatment [2, 5]. Reducing diagnostic delays is critical to reducing primary loss to follow-up and ongoing transmission of DR-TB [6–9]. While a reduction in time to diagnosis has been reported with Xpert MTB/RIF, there is less evidence that reducing time to treatment initiation (TTI) results in better treatment outcomes.

Despite improvements in diagnostic technology and access to care via decentralization of services, many patients diagnosed with MDR/RR-TB in South Africa continue to experience delays in treatment initiation and failure to link from testing to care [10]. Estimating the number of such patients is challenging, as two unlinked data sources are required. The number of diagnosed DR-TB patients is derived from centralized laboratory records, while the number who start DR-TB treatment is obtained from TB treatment registers (paper and electronic) kept at treatment sites.

In this study we compare the proportions of patients with MDR/RR-TB who successfully linked to care in two cohorts with different levels of Xpert MTB/RIF coverage: July 2011 to June 2012 (early Xpert MTB/RIF implementation, limited coverage) and July 2013 to June 2014 (late Xpert MTB/RIF implementation, full coverage). We then evaluate whether delays in treatment initiation have a subsequent impact on treatment outcomes.

## Methods

We conducted a retrospective medical register review to match adult patients diagnosed with laboratory-confirmed MDR/RR-TB, as reported to the City of Johannesburg Health Office (COJ), to treatment initiation records at the city’s four public sector DR-TB treatment sites between July 2011 and June 2014. Patients who transferred into Johannesburg after starting treatment in another district, province, or country were excluded from the analysis.

### Defining the early and late Xpert MTB/RIF cohorts

In order to determine the impact of Xpert MTB/RIF and decentralized DR-TB care on the proportion of persons diagnosed with RR-TB who initiated DR-TB treatment or time to treatment initiation, and whether this resulted in an improvement in DR-TB treatment outcomes, we defined two cohorts (e.g. early and late Xpert MTB/RIF implementation) and compared key TB indicators between the two cohorts.

South Africa’s National Health Laboratory Service (NHLS) rolled-out Xpert MTB/RIF to its national network of referral laboratories between 2011 and 2013. By May/June 2012 Xpert MTB/RIF implementation was 35% complete, and by July 2013 there was full (> 90%) coverage of Xpert MTB/RIF in the study area. We divided the study population into two 12-month time periods based on specimen collection date: an early/limited access Xpert MTB/RIF cohort from July 2011 to June 2012 (hereafter referred to as “early”), and an expanded/full access Xpert MTB/RIF cohort from July 2013 to June 2014 (hereafter referred to as “late”). The selection of these periods is consistent with what has been used in previous reports [2], and supported by data on Xpert MTB/RIF coverage from the NHLS for the COJ (Additional file 1: Figure S1).

### Data collection

The COJ Health Office, using a team of sub-district TB coordinators, coordinates the DR-TB response throughout the municipality, which includes both the central city and a large number of suburban neighborhoods and informal settlements surrounding it and has a population of approximately 4.4 million. The DR-TB response includes treatment at three decentralized (outpatient) hospital-based clinics and one centralized (inpatient) DR-TB hospital, according to the South African National TB guidelines [4, 11]. TB test results with indication of rifampicin drug resistance are communicated from the NHLS to the COJ where the patient is entered into a central electronic COJ register, alongside a DR-TB case registration number. The register captures first name, last name, date of birth, sex, tracing outcome, disease classification, diagnosis method, date of specimen collection, smear microscopy status, and patient address.

We identified our cohorts from the COJ register based on the specimen collection date and used NHLS data to verify the diagnosis method and specimen collection date. We enrolled all adult patients (18 years and older) who were diagnosed between July 2011–June 2012 or between July 2013–June 2014 and assigned a DR-TB case registration number by the COJ.

**Fig. 1:**
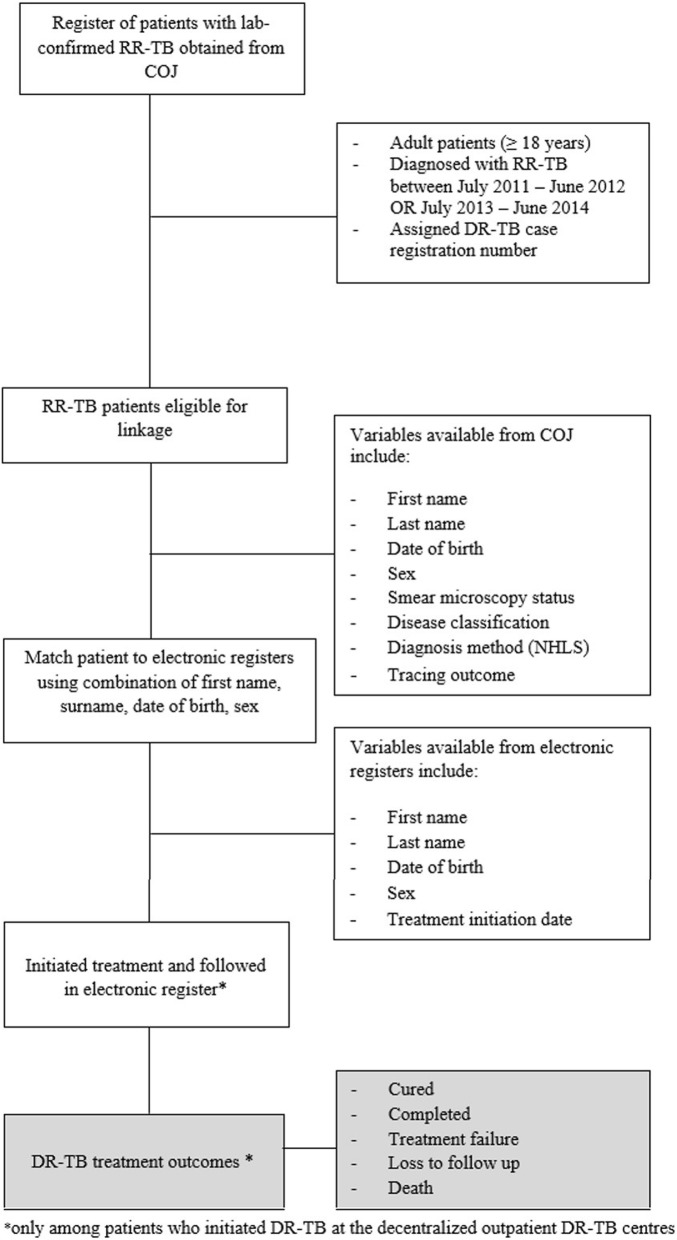
Schematic illustrating inclusion and retrospective follow-up of patients diagnosed with laboratory-confirmed RR-TB, as reported to the City of Johannesburg

We then matched patients from the COJ register to the treatment registers at the four DR-TB treatment sites (Fig. 1). Details of the matching and the validation of the matching algorithm have been described before [3]. Treatment start dates were obtained from treatment registers. For patients who were treated at the decentralized outpatient DR-TB centers, we also recorded the final treatment outcome assigned in the treatment registers, which allowed us to conduct a sub-analysis of outcomes as described below. Patients were followed for at least 24 months from the date of treatment initiation. Study staff worked closely with facilities and the COJ to verify and correct patient information (e.g. to correctly record DR-TB registration numbers in facility registers and query specific cases where diagnosis date or treatment initiation date preceded the sputum collection date) [3]. Medical records could only be queried for patients who initiated DR-TB treatment at one of the city’s four treatment sites. Patient information for those who did not initiate DR-TB treatment at one of the city’s four treatment sites could not be checked (e.g. at the diagnosing/referring facility).

### Classification of drug-resistance patterns

Patients diagnosed with rifampicin resistance by Xpert MTB/RIF with unknown susceptibility to isoniazid or second-line TB drugs were classified as having rifampicin resistant TB (RR-TB). When susceptibility test results for isoniazid and second-line drugs were available, patients were categorized as either rifampicin mono-resistant (RMR), multi-drug resistant (MDR-TB) (i.e. resistant to rifampicin and isoniazid) or extensively drug resistant (XDR-TB) (i.e. resistant to rifampicin, isoniazid and second-line TB drugs) [11, 12].

### Outcomes

#### Linkage to care

The primary outcome of interest was the proportion of patients diagnosed with MDR/RR-TB who initiated DR-TB treatment at one of the city’s four treatment sites within 6 months of specimen collection. Patients were followed from the specimen collection date until the earliest of treatment initiation (linkage), all-cause mortality, loss to care, transfer or relocation out of the city, or 6 months’ follow-up. Possible outcomes in this linkage to care analysis included death before linkage, moved or transferred out of the city, lost to follow-up (untraceable), failed to link to care or successfully traced and referred for treatment.

Descriptive statistics (frequencies, medians, and interquartile ranges) were used to present demographic and clinical characteristics present at diagnosis. The diagnosis method and diagnosis date were extracted from the COJ register and we present the median time from specimen collection to diagnosis date.

#### Impact of time to treatment initiation on final outcome of treatment

We defined time to treatment initiation as the difference between the specimen collection date and the date that DR-TB treatment was initiated. We compared median time to treatment initiation by disease classification (i.e. RR-TB, RMR, MDR-TB or XDR-TB) and diagnosis method using the student t test for parametric or Kruskal-Wallis for non-parametric data. Using the time to treatment initiation and the diagnostic method used, treatment initiation was categorized as delayed (deferred) or without delay (Table 1). In the sub-study, we also analyzed the impact of time to treatment initiation on final outcomes of treatment.

**Table 1:** Treatment initiation categorized as delayed (deferred) or without delay according to time to treatment initiation and diagnosis method

Diagnosis method	Without delay	Delayed (deferred)
Xpert MTB/RIF	≤ 7 days	> 7 days
Line probe assay (LPA)		
Positive smear	≤ 24 days	> 24 days
Negative/unknown	≤ 60 days	> 60 days
Phenotypic drug sensitivity testing (DST)	≤ 80 days	> 80 days

#### Final treatment outcomes

For the sub-study among patients who initiated DR-TB treatment at the three decentralized outpatient DR-TB centers, DR-TB outcomes were assigned in the case registers according to standard WHO definitions and 2013 WHO Reporting Framework for Tuberculosis as treatment success (cured and completed), failed, lost to follow-up, died, or transfer to another facility [12, 13]. Data from patients who transferred out or did not have an outcome assigned (i.e. missing) were excluded from this analysis, as no final treatment outcomes were available.

We identified patient characteristics at DR-TB initiation associated with treatment success (24 months after the DR-TB treatment start date) using log-binomial regression to estimate the relative risk and 95% confidence interval. We also identified predictors of on-treatment, all-cause mortality using Cox proportional hazards to estimate the hazard ratio and 95% confidence interval. Patients were followed from the DR-TB treatment start date until the earliest of all-cause mortality, lost to follow-up, treatment failure, treatment failure or 24 months’ follow-up. Variables in the univariate model that were significant at the 0.25 level along with other a priori identified characteristics (e.g. age, HIV status, gender etc.) were included in the multivariate model. The univariate and multivariate (adjusted) results are presented. Patients who initiated or were later referred for treatment at Sizwe Tropical Diseases Hospital, the centralized DR-TB inpatient treatment site for the province, were not included in this analysis as they had more extensive resistance (e.g. XDR or pre-XDR) or other reasons rendering them ineligible for decentralized DR-TB care.

All analyses were carried out using SAS version 9.3 (SAS Institute, Cary, North Carolina, USA). The study was a retrospective register review of routinely captured records and a waiver of informed consent was granted to retrospectively review these records. This study and the analysis of anonymized data was approved by the Human Research Ethics Committee (Medical) of the University of the Witwatersrand (Wits HREC M130601).

## Results

A total of 594 patients were included in the early cohort and 713 patients in the late cohort. The proportion of RR-TB patients diagnosed by Xpert MTB/RIF increased from 43.4% during the early cohort to 60.5% during the late cohort, with the remaining 33.9% diagnosed by line probe assay (LPA) despite guidelines calling for resistance to be identified by Xpert MTB/RIF. Most (62.6 and 76.4% for the early and late cohorts, respectively) patients did not have a smear microscopy result reported (Table 2).

**Table 2:** Demographic and clinical characteristics of patients who had a diagnosis of laboratory-confirmed RR-TB reported to the COJ for tracing between July 2011–June 2012 (*n* = 594) and July 2013–June 2014 (*n* = 713) and those included in the treatment outcomes sub-study (*n* = 537)

Characteristic		Early (*n* = 594)	Late (*n* = 713)	Included in sub-analysis (*n* = 537)
Gender	*n*, %			
Male		307 (51.6)	397 (55.6)	279 (51.9)
Female		287 (48.3)	316 (44.3)	258 (48.0)
Age, years	Median, IQR	34 (29–42)	37 (30–44)	36 (29–43)
< 30		162 (27.3)	161 (22.6)	138 (25.7)
30–45		308 (51.9)	376 (52.7)	282 (52.5)
45–60		105 (17.6)	159 (22.3)	106 (19.7)
≥ 60		19 (3.2)	17 (2.4)	11 (2.1)
DR-TB classification	*n*, %			
RR-TB by Xpert MTB/RIF		158 (26.6)	316 (44.3)	237 (44.1)
RIF mono-resistant TB		102 (17.2)	130 (18.2)	109 (20.3)
MDR-TB		320 (53.8)	262 (36.8)	191 (35.6)
XDR-TB		14 (2.4)	5 (0.7)	n/a
Sputum collection to diagnosis, days	Median, IQR	26 (7–36)	2 (1–7)	13 (7–28)
Diagnosis method from the COJ register	*n*, %			
Xpert MTB/RIF		258 (43.4)	431 (60.5)	337 (62.8)
Geno Type MTBDR plus line probe assay		281 (47.3)	242 (33.9)	172 (32.0)
Phenotypic drug sensitivity testing		30 (5.1)	2 (0.3)	6 (1.1)
Unknown		25 (4.2)	38 (5.3)	22 (4.1)
AFB smear microscopy	*n*, %			
Positive		144 (24.3)	119 (16.7)	94 (17.4)
Negative		78 (13.1)	49 (6.9)	44 (8.2)
Unknown		372 (62.6)	545 (76.4)	400 (74.4)
HIV status at initiation	*n*, %			
Positive		155 (26.1)	336 (47.1)	428 (79.7)
HIV positive on ART		n/a	n/a	351/428 (82.0)
HIV positive not on ART		n/a	n/a	77/428 (17.9)
HIV Negative		24 (4.0)	78 (10.9)	61 (11.4)
Unknown		415 (69.9)	299 (42.0)	48 (8.9)
Time on ART, months	Median, IQR			
ART initiation before TB treatment initiation (*n* = 171)		n/a	n/a	9.6 (3.1–27.4)
ART initiation after TB treatment initiation (*n* = 117)		n/a	n/a	0.5 (0.4–0.9)

*MDR-TB* Multi-drug resistant, *XDR-TB* Extensively drug resistant TB, *RR-TB* Rifampicin resistant TB, *AFB* Acid fast bacilli, *ART* antiretroviral therapy, *DR-TB* drug resistant TB, *RIF* rifampicin

Patients in the late cohort were slightly older and more likely to be male than those in the early cohort (median age of 37 years IQR 30–44 and 55.6% in the late cohort vs. 34 years IQR 29–42 and 51.6% in the early cohort). HIV prevalence in our early and late cohorts was lower than has been described nationally, with HIV prevalence of 26.1% in the early cohort vs. a national prevalence of 53.2% [14] and 47.1% in the late cohort vs. national 66% [2]. This may be due to the high number of patients in our cohorts with a HIV status missing, reflecting the limitations of routine data use [5, 14].

### Treatment referral and initiation

**Fig. 2:**
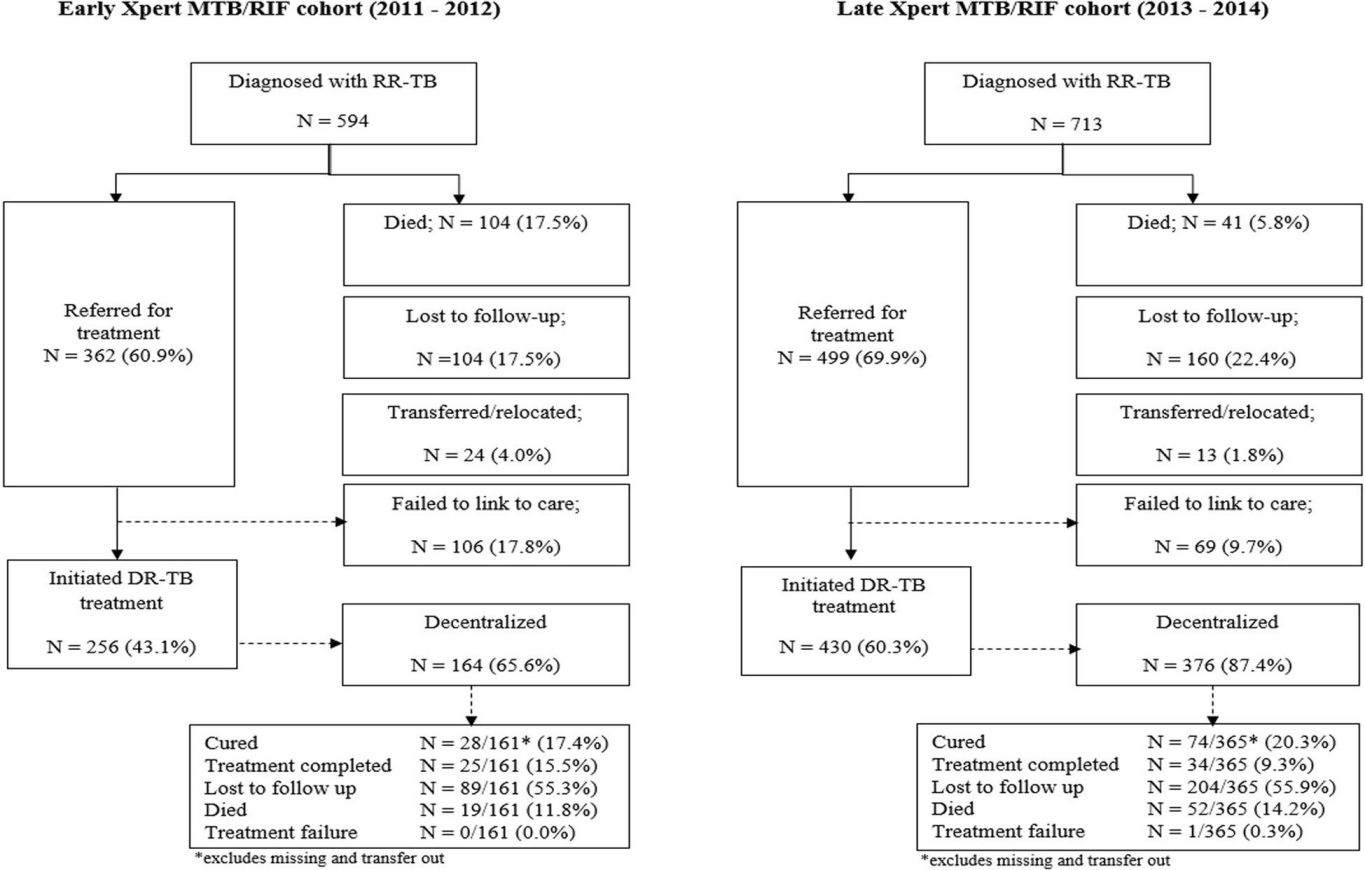
Treatment referral and initiation of patients who had a diagnosis of laboratory-confirmed RR-TB reported to the COJ for tracing between July 2011–June 2012 (*n* = 594) and July 2013–June 2014 (*n* = 713)

The total number of cases diagnosed at the four study sites increased from 594 in the early period to 713 in the late period, representing a 20% increase. In the early cohort, 60.9% (362/594) were successfully traced by COJ and referred for treatment. This increased to 69.9% (499/713) in the late cohort (Fig. 2). The proportion of patients diagnosed with RR-TB in COJ who initiated treatment at one of the four treatment sites within 6 months of diagnosis increased from 43.1% (256/594) in the early period to 60.3% (430/713) in the late period. The proportion who initiated at a decentralized (outpatient) TB facility, rather than the centralized inpatient facility, increased from 65.6 to 87.4% in the early and late cohorts, respectively.

### Time to diagnosis and treatment initiation

Median time from specimen collection to diagnosis decreased considerably from 26 days (IQR 7–36) in the early cohort to 2 days (IQR 1–7) in the late cohort. Median time from specimen collection to treatment initiation fell from 33 days (IQR 12–52) during the early cohort to 14 days (IQR 7–31) during the late cohort. Decreases in time to initiation varied by diagnostic test and disease classification, as shown in Table 3. The median time to treatment initiation dropped from 17 days (IQR 9–46) to 13 days (IQR 7–28) for patients diagnosed with Xpert MTB/RIF and from 38 days (IQR 23–51) to 15 days (IQR 7–36) for those diagnosed by LPA. The percentage of diagnosed patients who initiated treatment within 5 days, which is the national target increased from 6% (15/256) in the early cohort to 19% (82/430) in the late cohort [15].

**Table 3:** Median time from specimen collection to treatment initiation, by diagnostic method and disease classification (*n* = 686)

	Early cohort (*n* = 256)	Late cohort (*n* = 430)	*P* value
Diagnosis method			
Xpert MTB/RIF	17 (9–46) (*n* = 107)	13 (7–28) (*n* = 282)	< 0.001
GenoType MTBDRplus line probe assay	38 (23–51) (*n* = 129)	15 (7–36) (*n* = 129)	
Phenotypic drug susceptibility testing	81 (28–97) (*n* = 7)	n/a	
Disease classification			
RR-TB by Xpert MTB/RIF	13 (9–30) (*n* = 57)	10 (7–17) (*n* = 188)	< 0.001
RIF mono-resistant TB	48 (30–75) (*n* = 48)	13 (2–38) (*n* = 62)	
MDR-TB	34 (14–49) (*n* = 147)	22 (8–37) (*n* = 128)	
XDR-TB	42 (35–70) (*n* = 3)	36 (14–61) (*n* = 4)	

*MDR-TB* Multi-drug resistant, *XDR-TB* Extensively drug resistant TB, *RR-TB* Rifampicin resistant TB, *RIF* rifampicin

### Linkage to care and treatment initiation

Death before treatment initiation decreased from 17.5% (*n* = 104) in the early cohort to 5.8% (*n* = 41) in the late cohort; median time from specimen collection to death was 19 days (IQR 10–30) and 29 days (IQR 12–36) in these cohorts, respectively. Loss to follow up remained high at 17.5 and 22.4%, though the proportion of patients who were successfully traced but then failed to link to care decreased from 17.8 to 9.7% (Fig. 2). Median time from specimen collection to other reported outcome (lost to follow-up/untraceable or transferred out of the city/moved) was 14 days (IQR 8–34, *n* = 44) for the early and 24 days (IQR 9–57, *n* = 34) for the late cohort, respectively.

### Sub-study of outcomes and patient characteristics

Five hundred thirty seven patients initiated treatment at one of the three decentralized facilities, a number that excludes those who initiated at the centralized inpatient facility, transferred out, did not have an outcome assigned (i.e. missing) or had more extensive resistance (i.e. XDR or pre-XDR TB). Roughly half were 30–45 years old (52.5%) and male (51.9%), and 62.8% were diagnosed by Xpert MTB/RIF. Of those who initiated treatment, 79.7% (428/537) were HIV positive, with most on ART (82.0%, 351/428), which was comparatively higher than that reported for the early and late cohorts and reflects the limitations of using routine data from two unlinked data sources (e.g. 42–70% of patients in the COJ register had an unknown HIV status compared to < 10% in the DR-TB treatment register) (Table 2).

Outcomes could be evaluated for 62.8% (161/256) of patients who initiated during the early Xpert MTB/RIF period and 84.9% (365/430) of those who initiated in the late Xpert MTB/RIF period. Treatment success was similar between the two periods (32.9% vs. 39.6% for the early and expanded Xpert MTB/RIF cohorts, respectively). Despite decentralization and Xpert MTB/RIF implementation, all-cause on-treatment mortality (11.8% vs. 14.2%) and loss to follow-up (55.3% vs. 55.9%) remained high for both the early and late cohorts (Fig. 2).

The only factor associated with treatment success was pattern of drug resistance (RR-TB by Xpert MTB/RIF vs. MDR-TB; aRR 4.31 95% CI 2.55–7.29 and RMR vs. MDR-TB; aRR 4.46 95% CI 2.07–7.95) (Table 4). There was no association between time to treatment initiation (i.e. delayed treatment initiation vs. without delay) and treatment success.

**Table 4:** Factors associated with treatment success by 24 months among patients who were diagnosed with RR-TB between July 2011–June 2012 or between July 2013–July 2014 and who initiated DR-TB treatment in COJ (*n* = 537)

	n/N	%	RR	95% CI	Adjusted RR	95% CI
Gender						
Female	75/258	29.1	Ref	Ref	Ref	Ref
Male	86/279	30.8	1.09	(0.79–1.49)	1.01	(0.74–1.41)
Age, years						
< 30	31/138	22.5	Ref	Ref	Ref	Ref
30–45	93/282	32.9	**1.56**	**(1.02–2.37)**	1.46	(0.94–2.25)
≥ 45	37/117	31.6	1.46	(0.89–2.38)	1.43	(0.86–2.37)
DR-TB classification						
RR-TB by Xpert MTB/RIF	96/237	40.5	**4.36**	**(2.60–7.30)**	**4.31**	**(2.55–7.29)**
RIF mono-resistant TB	47/109	43.1	**4.76**	**(2.70–8.36)**	**4.46**	**(2.07–7.95)**
MDR-TB	18/191	9.4	Ref	Ref	Ref	Ref
HIV status at initiation						
HIV negative	14/61	22.1	0.58	(0.32–1.05)	0.75	(0.47–1.20)
HIV positive on ART	120/351	33.4	Ref	Ref	Ref	Ref
HIV positive not on ART	16/77	19.5	0.6	(0.36–1.02)	0.73	(0.43–1.25)
HIV status unknown	11/48	22.9	0.64	(0.33–1.21)	0.76	(0.43–1.24)
Registration year						
2011	19/55	34.5	Ref	Ref	Ref	Ref
2012	33/108	30.6	0.88	(0.50–1.56)	0.88	(0.49–1.56)
2013	49/166	29.5	0.81	(0.47–1.37)	0.74	(0.43–1.28)
2014	60/211	28.4	0.84	(0.50–1.41)	0.83	(0.49–1.41)
Time to treatment initiation						
Without delay	70/186	37.6	Ref	Ref	Ref	Ref
Delayed (deferred)	85/332	25.6	0.97	(0.71–1.33)	0.94	(0.66–1.33)

*RR* Relative Risk, *CI* Confidence interval, *RR-TB* Rifampicin resistant TB, *MTB* Mycobacterium tuberculosis, *MDR-TB* Multi-drug resistant tuberculosis, *XDR-TB* Extensively drug-resistant tuberculosis; *DR-TB* drug resistant TB, *ART* antiretroviral therapy, *RIF* rifampicin *p* < 0.05 bold

We also identified predictors of all-cause mortality after DR-TB treatment initiation (Table 5). Compared to HIV positive patients on ART, HIV negative patients were less likely to die during the 24 months of follow-up on treatment (aHR 0.13 95% CI 0.02–0.10). Delayed (deferred) treatment initiation was not identified as a predictor of all-cause mortality (delayed vs. without delay; aHR 0.91 95% CI 0.53–1.56).

**Table 5:** Unadjusted and adjusted predictors of mortality among patients who were diagnosed with RR-TB between July 2011–June 2012 or between July 2013–July 2014 and who initiated DR-TB treatment in COJ (*n* = 537)

	n/N	%	HR	95% CI	Adjusted HR	95% CI
Gender						
Female	34/258	13.2	Ref	Ref	Ref	Ref
Male	33/279	11.8	0.78	(0.48–1.26)	0.74	(0.44–1.22)
Age, years						
< 30	17/138	12.3	Ref	Ref	Ref	Ref
30–45	37/283	13.1	0.92	(0.52–1.64)	0.72	(0.39–1.31)
≥ 45	13/119	10.9	0.75	(0.36–1.58)	0.68	(0.32–1.47)
DR-TB classification						
RR-TB by Xpert MTB/RIF	41/237	17.3	1.16	(0.58–2.33)	1.03	(0.50–2.13)
RIF mono-resistant TB	15/109	13.8	0.87	(0.39–1.97)	0.65	(0.33–1.81)
MDR-TB	11/191	5.8	Ref	Ref	Ref	Ref
HIV status at initiation						
HIV negative	1/61	1.6	**0.14**	**(0.02–1.01)**	**0.13**	**(0.02–0.10)**
HIV positive on ART	53/351	33.6	Ref	Ref	Ref	Ref
HIV positive not on ART	10/77	12.1	1.49	(0.76–2.94)	1.49	(0.75–2.96)
HIV status unknown	3/48	6.3	0.38	(0.09–1.54)	0.36	(0.09–1.50)
Registration year						
2011	6/55	10.9	Ref	Ref	Ref	Ref
2012	11/108	10.2	0.89	(0.33–2.40)	0.88	(0.34–2.59)
2013	21/166	21.7	1.3	(0.55–3.36)	1.32	(0.52–3.33)
2014	29/211	13.7	1.45	(0.59–3.51)	1.37	(0.55–3.41)
Time to treatment initiation						
Without delay	31/186	16.7	Ref	Ref	Ref	Ref
Delayed (deferred)	35/332	10.5	0.89	(0.54–1.45)	0.91	(0.53–1.56)

*HR* Hazard Ratio, *CI* Confidence interval, *RR-TB* Rifampicin resistant TB, *MTB* Mycobacterium tuberculosis, *MDR-TB* Multi-drug resistant tuberculosis, *XDR-TB* Extensively drug-resistant tuberculosis; *ART* antiretroviral therapy, *RIF* rifampicin, *DR-TB* drug resistant TB *p* < 0.05 bold

## Discussion

Tuberculosis continues to kill more people worldwide than any other infectious disease [16]. The Global Burden of Disease study 2015 concludes that strengthening health systems for early TB case detection and improved quality of diagnosis and treatment are essential to addressing this epidemic [17]. In two cohorts of DR-TB patients diagnosed in Johannesburg, South Africa in 2011 and 2013, we found that the introduction of Xpert MTB/RIF was associated with a substantial decrease in time to treatment initiation and with an increase in the proportion of patients who linked to care and initiated appropriate DR-TB treatment [2, 5]. Our study demonstrated an additional benefit, observed during the late period, which was a significant reduction in pre-treatment mortality.

We found that the number of people diagnosed with DR-TB within the COJ between 2001 and 2012 and 2013–2014 who initiated appropriate treatment within 6 months of diagnosis increased from 43.1 to 60.3%. Despite this large increase, some 40% of patients with a reported diagnosis of RR- or MDR-TB did not start treatment within 6 months, at least within the City of Johannesburg municipality. While a small proportion likely did start treatment outside the city, there remained a major gap in moving patients from diagnosis to treatment, despite the policy changes.

The increase in treatment initiation is similar to what was observed in a nationwide retrospective cohort, which reported 55 and 63% in 2011 and 2013 respectively [2, 5] and possibly reflects the national strategy for Xpert MTB/RIF implementation across districts or provinces, under which priority for pilot implementation or research sites in high burden districts. Our results also showed that the proportion of patients initiating appropriate DR-TB treatment within 5 days of being diagnosed increased from 6 to 19%, still four fifths of patients were not managed according to the National Department of Health recommendation to initiate DR-TB treatment within 5 days of being diagnosed [15].

Overall, time to treatment initiation decreased from a median of 33 to 14 days in the early and late cohorts, respectively. Among patients diagnosed by Xpert MTB/RIF, time to treatment initiation decreased from 17 to 13 days. Our observed time to treatment initiation is much lower than that reported by a national cohort study based on data from the national RR-TB register, which was 44 and 22 days [2]. This may reflect true variation by geographic region in South Africa or simply limitations of the national register, as only 53% of the 1448 new RR-TB patients who received treatment were recorded in the national register [2], but it may also reflect differences in the model of care. The majority (78%; 540/686) of patients in our cohorts initiated treatment at a decentralized, outpatient DR-TB center, whereas the national cohort study included patients from both decentralized, outpatient and centralized, inpatient treatment facilities. The model of care has been shown to contribute to a decrease in time to treatment initiation, with outpatient sites demonstrating significantly lower time to treatment initiation compared to centralized, inpatient sites [3].

Pre-treatment mortality declined significantly from 17.5 to 5.8% between the early and late cohorts. As only 60.9% of patients in the late cohort were diagnosed using Xpert MTB/RIF, further expansion of Xpert MTB/RIF may make further reductions in pre-treatment mortality possible. Results suggest that the quality of tracing and success of linkage to care improved between the early and late periods of our study. The proportion of patients who were successfully traced and referred for treatment increased by 9%, while the proportion of patients contacted and referred for treatment who failed to link to care in the expanded Xpert MTB/RIF cohort was roughly half of that observed during the early Xpert MTB/RIF cohort (17.8% vs. 9.7%). These tracing efforts may have translated into improved pre-treatment outcomes (i.e. more patients initiating appropriate DR-TB treatment and reducing mortality in the pre-treatment period). Not only was pre-treatment mortality significantly reduced between the early and late cohorts (17.5 to 5.8%), but the time from sputum collection to death significantly increased from 19 to 29 days. The reduction in mortality may be associated with a reduction in time to treatment initiation observed during the late period. Time from specimen collection to the date a patient was considered lost to follow-up, untraceable, or transferred out of the city also increased from a median of 14 to 24 days. We speculate that tracing efforts improved during the late Xpert MTB/RIF period, causing the sickest patients to be diagnosed and started on treatment earlier while those who were less symptomatic failed to link to care and went on to die later.

Despite improved linkage to care and a reduction in pre-treatment mortality, pre-treatment loss to follow up remained high (17.5 and 22.4%). Interventions such as the implementation of decentralized TB treatment and Xpert MTB/RIF thus do not appear sufficient to address alarmingly high rates of pre-treatment loss [3]. TB services could benefit from the implementation of a unique patient identifier so that health care workers can focus tracing efforts on patients who are “truly lost” and not waste efforts or resources on patients who have moved or transferred out of the city and initiated care at another facility, in the same or a different province.

We found no difference in on-treatment mortality (11.8% vs. 14.2%) and loss to follow-up (55.3% vs. 55.9%) when comparing the early and the late cohorts. Consistent with what others have reported, a reduction in time to treatment initiation did not result in improved treatment outcomes [18–24]. Prior studies have found no significant difference in on-treatment mortality between diagnosis by Xpert MTB/RIF and other diagnostic methods [8, 25–27]. As others have reported, despite reductions in time to treatment initiation, early treatment outcomes remain poor with high rates of death and loss from care [28].

HIV status was the only factor associated with mortality by 24 months. HIV negative patients were less likely to die compared to HIV-positive patients on ART (aHR 0.13 95% CI 0.02–0.10). HIV prevalence in the late cohort was lower than has been described nationally among patients with rifampicin resistant TB (47.1% vs. 66%) [2], but higher in the sub-analysis (79.7%) as the data was more complete.

Results should be considered in light of the study limitations. First, Xpert MTB/RIF and decentralization of treatment in the COJ were rolled out in the same period. We cannot attribute our findings to either specific change, only to the combination of both. We did not have any data to explain differences in program performance during the early and late cohort periods. To address this, we allowed a 12-month period (July 2012 – June 2013) between the two comparison cohorts to offset differences in program activity and resources as Xpert MTB/RIF and decentralized care were rolled-out across the different facilities. Second, the sub-analysis was limited to patients who initiated DR-TB treatment at one of the three decentralized outpatient DR-TB centers, thus potentially excluding those more likely to have worse outcomes (e.g. XDR, pre-XDR or those with poor clinical status). Third, treatment outcomes were missing for 37.2% of sub-study patients, and those without reported outcomes may have been more likely to have been lost to follow-up or died than those whose outcomes we observed. Data on HIV and ART status and time on ART were missing for patients who did not initiate DR-TB treatment. We also believe that among those with a diagnosis of RR-TB reported to the COJ, the proportion co-infected with HIV may be underestimated due to missing data. Nationally, for the period 2013, 13% of patients with RR-TB had a missing HIV status, and this varied (9–22%) across the nine provinces [2]. This is substantially lower than what we report (42%) for the same period, and highlights the limitations of routine data. Some patients may have initiated treatment outside of the city, but existing tracing systems did not allow this to be determined. Finally, we speculate that mortality was underreported, as some patients who did not complete treatment and were reported as lost to follow-up or without an outcome likely died.

## Conclusion

With South Africa’s expansion of Xpert MTB/RIF coverage and treatment decentralization, both pre-treatment mortality and time to treatment initiation for DR-TB fell markedly. Xpert MTB/RIF resulted in an increase in the proportion of patients diagnosed with RR-TB who initiated treatment. These improvements did not, however, correspond to reductions in pre-treatment loss to follow-up or better DR-TB treatment outcomes and on-treatment mortality and loss to follow-up remained high.

Since rapid initiation of DR-TB treatment is key to controlling TB, patients who are diagnosed with TB who do not initiate treatment (i.e. pre-treatment loss to follow-up) represent an important failing in the provision of care [6]. This group, who should be targeted because of their increased risk of mortality and TB transmission to others, are not included in routine reporting by national treatment programs, thus overestimating program effectiveness [6, 29]. Programs should invest in novel ways to reduce pre-treatment loss to follow-up. Innovative approaches such as a mhealth linkage program or facility-based linkage officers should be explored to document pre-treatment loss to follow-up and improve linkage to care for DR-TB patients.

## Additional file

Additional file 1: **Figure S1.** Total number of Xpert MTB/RIF tests performed between 2011 and 2014 (source: NHLS). (JPG 43 kb)

## Abbreviations

aHR: Adjusted hazard ratio
aRR: Adjusted relative risk
ART: Antiretroviral therapy
COJ: City of Johannesburg
DR: Drug resistant
HIV: Human immunodeficiency virus
LPA: Line probe assay
MDR: Multi-drug resistant
MTB: Mycobacterium tuberculosis
NDOH: National Department of Health
NHLS: National Health Laboratory Service
RIF: Rifampicin
RMR: Rifampicin mono-resistant tuberculosis
RR-TB: Rifampicin-resistant tuberculosis
TB: Tuberculosis
TTI: Time to treatment initiation
USA: United States of America
WHO: World Health Organization
XDR: Extensively drug resistant
