# Supplementary figures and images for: Impact of Xpert MTB/RIF and decentralized care on linkage to care and drug-resistant tuberculosis treatment outcomes in Johannesburg, South Africa
Source: BMC Health Serv Res. 2018 Dec 17;18:973. doi: 10.1186/s12913-018-3762-x (PMC6296148; doi:10.1186/s12913-018-3762-x)

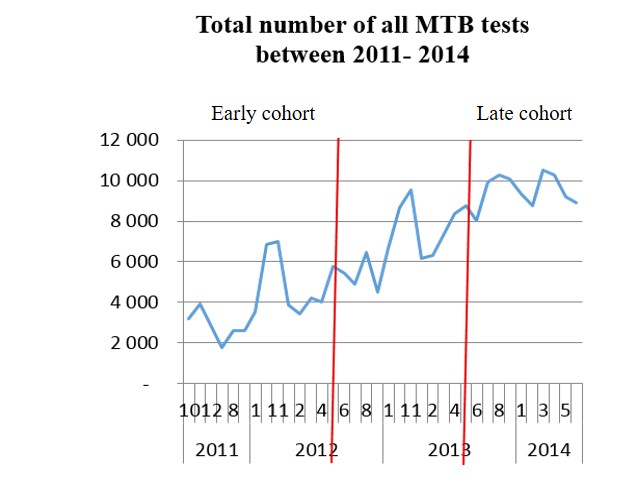

Supplement: Supplementary file 1 — Figure S1. Total number of Xpert MTB/RIF tests performed between 2011 and 2014 (source: NHLS). (JPG 43 kb) [file 12913_2018_3762_MOESM1_ESM.jpg]
